# Enhancement of Lipid Metabolism and Hepatic Stability in Fat-Induced Obese Mice by Fermented* Cucurbita moschata* Extract

**DOI:** 10.1155/2018/3908453

**Published:** 2018-03-13

**Authors:** Md. Akil Hossain, Seung-Jin Lee, Na-Hye Park, Biruk Tesfaye Birhanu, Abraham Fikru Mechesso, Ji-Yong Park, Eun-Jin Park, Sam-Pin Lee, Sun-Joo Youn, Seung-Chun Park

**Affiliations:** ^1^College of Veterinary Medicine, Kyungpook National University, Daegu 702-701, Republic of Korea; ^2^Veterinary Drugs & Biologics Division, Animal and Plant Quarantine Agency (APQA), 177 Hyeoksin 8-ro, Gimcheon-si, Gyeongsangbuk-do 39660, Republic of Korea; ^3^Department of Food Science and Technology, Keimyung University, Daegu 704-701, Republic of Korea; ^4^The Aramfarm Co., Ltd, Gyeongsan 38497, Republic of Korea

## Abstract

The aim of this study was to evaluate the potentials of fermented* Cucurbita moschata* extract (FCME) in the treatment of obesity and nonalcoholic fatty liver disease (NAFLD). Five-week-old male C57BL/6 mice were assigned to 6 groups and treated for 8 weeks by feeding the normal diet (ND) and high fat diet (HFD) with and without FCME. Changes in body weight gain and consumption of feed and water were recorded. Major organs, adipose tissues, and blood samples were collected after the experimental period. The serum lipid profile, histological features of liver and adipose tissues, and mRNA expression of different adipogenic/lipogenic genes from liver tissue were evaluated. The supplementation of FCME in HFD significantly prevented HFD-induced increment of bodyweight. The adipose tissue mass, liver enzymes, and plasma lipids were also reduced significantly (*p* < 0.05) by the consumption of FCME. The mRNA expressions of adipogenic/lipogenic genes (PPAR*γ*, C/EBP*α*, C/EBP_*β*_, C/EBP*γ*, and SREBP-1C) in FCME-treated obese mice were considerably (*p* < 0.05) suppressed. FCME showed its antiobesity potential by suppressing the body weight gain and by modulating the plasma lipids and liver enzymes through the regulation of adipogenic/lipogenic transcriptional factors. Fermented* Cucurbita moschata* could be an opportunistic agent in controlling obesity and fatty liver changes.

## 1. Introduction

Obesity is a major health concern of the 21st century, serving as a risk factor for various diseases, including diabetes, coronary artery disease, arthritis, and hypertension [1]. Obesity is associated with a spectrum of liver abnormalities, known as nonalcoholic fatty liver disease (NAFLD). NAFLD has become an important public health problem due to its high pervasiveness, association with serious cardiometabolic abnormalities, and potential progression to severe liver diseases [2]. Currently, NAFLD treatments include exercise, balanced diet, and medications like metformin, fibrates, statins, orlistat, lorcaserin, and sibutramine. Although, these drugs are reported for several adverse effects or contraindications, there is still no consensus on the most effective drug therapy [3]. Therefore, new candidates with high efficiency and no or little side effects are immediately required to treat NAFLD and obesity. In the last decade, nutraceuticals and functional foods have attracted great scientific interest, with a number of clinical trials and animal studies addressing their potential roles in blood lipid control [4]. The beneficial hypocholesterolemic and/or antioxidant effects of two plant-derived substances, N-acetylcysteine (NAC) and sesame oil, have been reported previously [5]. One of the opportunistic natural agents, namely, pumpkin, has received substantial considerations recently because of the nutritional and health-enhancing value of the seeds as well as the polysaccharides from the fruit [6].

Pumpkin* (Cucurbita moschata)* is rich in polysaccharides, vitamins, mineral salts, carotene, and other substances beneficial to health [10]. Pumpkin seeds were used by Native Americans to treat intestinal infections, which finally led the United States Pharmacopoeia to list pumpkin seeds as an official medicine for parasite elimination from 1863 to 1936. Preliminary investigations showed that a pumpkin-rich diet could reduce blood glucose, and the active polysaccharides from the pumpkin fruit could evidently increase the serum insulin levels, reduce the levels of blood glucose, improve glucose tolerance, and hence be developed as new antidiabetic agent [10].

Recent evidence suggests that high serum cholesterol concentration can be treated using a different factor, the gut microbiota. Lactobacilli are one of the vital health-enhancing microbiota which is frequently found as probiotics in cultured milks, infant foods, and different pharmaceutical preparations. One beneficial effect that has been suggested to result from human consumption of probiotic lactic acid bacteria (LAB) is a reduction in serum cholesterol levels [11]. One of the probiotic LAB,* Lactobacillus plantarum*, is also reported for having hypocholesterolemic effects in different studies [12]. It was demonstrated in previous study that* Bacillus subtilis* has hypoglycemic and antilipidemic activities [13]. Several studies have reported that probiotic-fermented food shows hypolipidemic effects [14]. Furthermore, fermentation significantly improves the functional properties of pumpkin products [10].

Thus, the current study was designed to evaluate the protective effect of fermented* Cucurbita moschata* extract against obesity and NAFLD in a high fat diet-induced obese mice model and to reveal the possible mechanism underlying to this assumed effect. Moreover, the safety profile of this fermented extract was assessed* in vivo* and the functional compounds were identified. To our knowledge, this is the first report of using fermented* Cucurbita moschata* extract to control the obesity and NAFLD.

## 2. Methods

### 2.1. Chemicals and Reagents

Garcinia extract containing ≥ 600 mg/g hydroxycitric acid (HCA) was obtained from Unicorn Natural Products Private Limited (Hyderabad, India). HPLC grade formic acid and acetonitrile were purchased from Honeywell Burdick & Jackson (Ulsan, South Korea).

### 2.2. Fermentation of* Cucurbita moschata*

Peeled* Cucurbita moschata* (pumpkin) was purchased from Aram farm Co. (Yeongcheon, South Korea) and then homogenized with two volumes of water. The pumpkin paste was fortified with 3% glucose and 1.5% monosodium glutamate to prepare 2.5 L culture medium, followed by sterilization at 121°C for 15 min.* Bacillus subtilis* HA was cultured in nutrient broth for 1 day. Cells were harvested by centrifuge at 22,500 ×g for 15 min and then washed with sterilized saline solution. The cell suspension of 10 times concentration was prepared to be inoculated with 1% level. The submerged culture was performed with 5 L jar fermentor (Fermentex Co. Ltd., Cheongwon, South Korea) in the condition of 450 rpm and air supply of 2.5~3 L/min at 42°C for 2 days. For the second fermentation using* Lactobacillus plantarum* EJ2014, starter culture grown in MRS broth for 1 day at 30°C was inoculated in the first fermented culture broth with 1% level. The mixed culture was cultivated by slightly shaking with 100 rpm at 30°C for 3 days. Final culture broth obtained by mixed fermentation using* B. subtilis* and* L. plantarum* was freeze-dried using a freeze dryer (Ilshinbiobase Co. Ltd., Docheon, South Korea) and preserved in 4°C refrigerator.

### 2.3. Animal Experimental Design

Five-week-old male C57BL/6 mice of 19–21 g were purchased from Orient Co. Seoul, South Korea (Charles River Technology). The animals were maintained at 20–25°C temperature, 55 ± 10% relative humidity, and 12 h light/dark cycle under specific-pathogen-free condition. Feed and water were given ad libitum. After 1 week of acclimatization, the mice were randomly assigned to 6 groups (10/group) as follows: control (normal diet, AIN-76A #100000, Dyets Inc., Bethlehem, PA, USA), high fat diet (HFD) containing 40% beef tallow modified AIN-76A purified rodent diet (#101556, Dyets Inc., Bethlehem, PA, USA), HFD with 0.3% HCA (in Garcinia extract), and HFD with 0.1%, 0.3%, 0.5% fermented* Cucurbita moschata* extract (FCME). HFD was fed for 8 weeks to induce the hyperlipidemia and obesity in mice. The composition of ND and HFD are presented in Supplementary Table 1. The animal experimental protocols were approved by the Institutional Animal Care and Use Committee of Kyungpook National University (approval number KNU 2011-1). The changes in body weight, feed, and water intake were recorded routinely. At the end of the experiment, blood was collected from the hearts of the mice under anesthesia. After euthanasia, kidney, liver, spleen, and the adipose tissues (subcutaneous, mesenteric, perirenal, and epididymal) were immediately collected, weighted, and preserved.

### 2.4. Biochemical Analysis of Serum

After overnight fasting, mice were anesthetized with diethyl-ether, and blood samples were collected from the hearts of mice into serum extraction tubes and allowed to coagulate properly. The tubes were centrifuged at 1500 ×g for 10 min at 4°C, and the serum was collected immediately. Biochemical examinations were performed for serum concentrations of total cholesterol (TC), high density lipoprotein (HDL), triglycerides (TG), and free fatty acid. Low density lipoprotein (LDL) was calculated by subtracting HDL from TC, and the atherogenic index (AI) was calculated as TC − HDL/HDL. These two formulas for calculating the LDL and AI are stated in many articles including in the article of Reza et al. [15]. Aspartate aminotransferase (AST) and alanine aminotransferase (ALT) were measured to determine the liver function.

The endocrine peptides such as ghrelin, leptin, insulin, and GIP (glucose-dependent insulin-releasing polypeptide) were also measured. All biochemical examinations were conducted by using commercially available ELISA kits. The employed ELISA kits of ALT, AST, free fatty acid, HDL, and TC were from Sigma-Aldrich Co. LLC. (St. Louis, MO 63103, USA); TG and insulin from abcam (Cambridge CB4 OF4, United Kingdom); ghrelin and leptin from EMD Millipore Corporation (Missouri 63304, USA); and GIP from Cusabio (Hubei 430206, China).

### 2.5. Histopathological Study of Liver Tissue

Liver tissues were fixed in 10% buffered formaldehyde. Tissue sections (5 *μ*m) were stained with Ehrlich's hematoxylin and eosin (H&E) and examined for the severity of hepatic steatosis as described previously [15].

### 2.6. Determination of Adipocyte Size

Adipocyte sizes in mice's adipose tissue were measured in paraffin-embedded tissue. Briefly, samples of mesenteric, subcutaneous, epididymal, and perirenal adipose tissue were fixed in 10% formalin and then embedded in paraffin. Tissue sections were cut into 5 *μ*m with a microtome (Leica, Germany) and mounted on Superfrost/Plus Microscope Slides. After being air-dried, they were stained with Ehrlich's hematoxylin and eosin and photographed at 100x magnification. At least two fields per slice and six slices per fat mass were analyzed for determining adipocyte size. The same region of the fat pad was used for all animals to minimize cell size variation due to differences in anatomical location.

### 2.7. Extraction of RNA from Liver Tissue

Frozen liver tissue was cut into slices and about 80 mg of the tissue was immediately transferred to an eppendorf tube. One milliliter of Trizol reagent was added and the tissue was homogenized periodically with IKA T10 basic Homogenizer (Seoul, South Korea) by keeping the tube on ice. RNA extraction was performed according to the protocol for the Trizol reagent. The resulting solution was diluted 100-fold, purity was confirmed, and RNA concentration (*μ*g/mL) was calculated by measuring absorbance at 260 and 280 nm using a U-2800 spectrophotometer (Hitachi High Technologies, Tokyo, Japan).

### 2.8. Real-Time Quantitative PCR

cDNA was synthesized from 100 ng of RNA by using RNA to cDNA EcoDry Premix (Oligo dT) (Clontech Laboratories, Inc., Seoul 153-779, South Korea) according to the supplied protocol. The expression of target genes was detected by performing real-time PCR with the primers listed in Supplementary Table 2. To each PCR tube (TLS0851, Bio-Rad Laboratories Inc., Herts HP2 7DX, United Kingdom), 12.5 *μ*L of SYBR Select Master Mix for CFX (Applied Biosystems, Foster City, CA 94404, USA), 1 *μ*L of forward primer (10 pmol), 1 *μ*L of reverse primer (10 pmol) of target gene, 9.5 *μ*L of RNase-free water, and 1 *μ*L of cDNA were added, mixed by pipetting, and spined down. The real-time PCR reaction was accomplished in CFX96 Touch™ Real-Time PCR Detection System (Bio-Rad Laboratories Inc., Irvine, CA 92618, USA) with 35 cycles of denaturation at 94°C for 30 s, annealing at 58°C for 45 s, and elongation at 72°C for 30 s. mRNA expressions were normalized using *β*-actin. All mRNA expressions were expressed in relation to the average expression of the ND group (100%).

### 2.9. Acute Oral Toxicity

Ten female Sprague-Dawley rats that were 8 weeks old were obtained from Orient Bio Inc. (Gyeonggi-do, South Korea). Animals were maintained in standard conditions as stated in Animal Experimental Design. The acute toxicity study was performed by slightly modifying a previously reported method [16]. Rats were randomly assigned to control and test groups (5 rats in each group). A single dose (2000 mg/kg of body weight) of FCME was administered intragastrically according to OECD test guideline 423. The animals were observed for abnormal signs and symptoms for first 12 h after the administration of FCME. They were also observed daily for 2 weeks. The changes in body weight, feed, and water intake were measured twice a week for 14 days. At the end of the experiment, animals were sacrificed, and major organs were collected and inspected for gross lesions.

### 2.10. Compound Profile Analysis of FCME

LC-MS analysis of FCME was accomplished by Agilent 1100 series LC system (Agilent Technologies, Santa Clara, CA) equipped with an Agilent 1946B mass selective detector (MSD) (Agilent Technologies, Santa Clara, CA). A Pursuit XRs C18 (2.0 mm × 150 mm, 3 *μ*m) column was utilized at a flow rate of 0.2 mL/min at 40°C. The mobile phase consists of A (0.1% formic acid in water) and B (0.1% formic acid in acetonitrile). The run time for each sample was 60 min, and the mobile phase started with 95% “A” and 5% “B” in the first 2 min; then “A” was decreased gradually to 0% until the 50th min and held for an additional 5 min. Thereafter, the proportion of “A” was increased to 95% within 5 min. The compounds were analyzed in selected ion monitoring (SIM) mode.

GC/MS analysis of FCME was performed by Kyungpook National University Center for Scientific Instruments and carried out using a HP 6890 Plus GC gas chromatograph with a (MSD)—HP 5973 MSD mass selective detector (Hewlett-Packard). Samples were diluted 1 : 1000 (v : v) with HPLC grade dichloromethane. Aliquots of the sample (1 *μ*L) were injected into an HP-5 column. The GC oven temperature was set at 50°C for 4 min, increased to 280°C at a rate of 4°C/min, and held at the final temperature for 2 min. Velocity of the He carrier gas (99.99%) was 0.7 mL/min. Quantitative analysis was performed using the area normalization method.

### 2.11. Statistical Analysis

Data are presented as mean ± standard error of three replicate assays. Analysis of variance (ANOVA) and *F*-test were performed and *p* values of less than 0.05 were considered to be statistically significant.

## 3. Results

### 3.1. Effect of FCME on Feed and Water Consumption and Body Weight Gain

**Figure 1:**
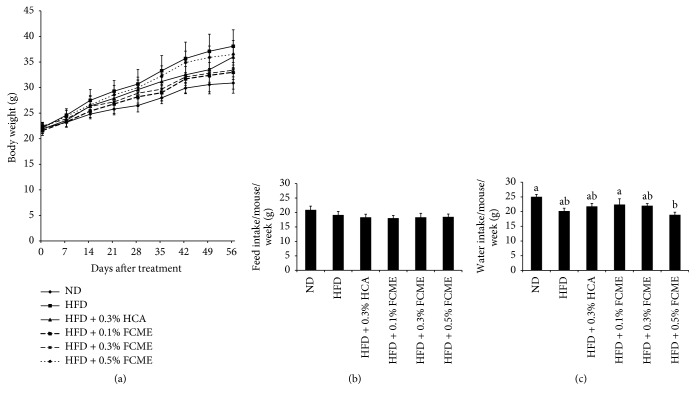
Effect of fermented* Cucurbita moschata* extract (FCME) on (a) body weight gain, (b) feed intake, and (c) water intake in high fat diet-fed C57BL/10 male mice for 8 weeks. Results are expressed as mean ± SE of 10 mice in each group. Values not having the same superscript letter are significantly different (*p* < 0.05) by ANOVA.

The body weight of HFD-fed mice increased significantly compared to other groups within the experimental period (Figure 1(a)). Feed intake and water intake were not affected significantly in different groups of animal (Figures 1(b) and 1(c)). The FCME plus HFD-fed mice showed a reduced body weight gain better than HCA group. It is demonstrated that there were no significant differences in the weight of kidney and spleen among different groups (Table 1). The weights of liver and adipose tissues (subcutaneous, mesenteric, perirenal, and epididymal) were increased considerably in HFD-fed mice compared to mice of ND group. The weights of liver and adipose tissues were noticeably lowered in 0.1% and 0.3% FCME-supplemented HFD-fed mice compared to mice fed only HFD.

### 3.2. Effect of FCME on Serum Lipids

**Figure 2:**
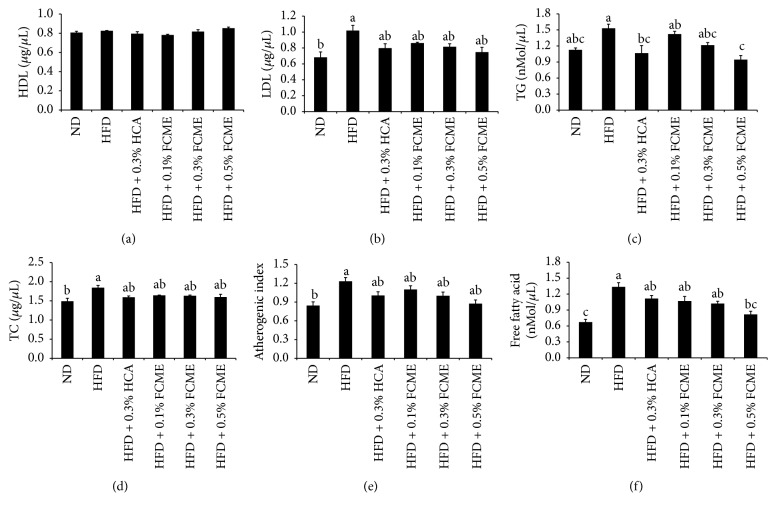
Changes of serum lipids in mice fed with normal diet; and high fat diet with or without 0.1%, 0.3%, and 0.5% of fermented* Cucurbita moschata* extract (FCME) for 8 weeks. (a) HDL, (b) LDL, (c) TG, (d) TC, (e) AI, and (f) FFA. Different superscript letters are significantly different (*p* < 0.05) by ANOVA.

The impact of FCME on serum lipid profile is presented in Figure 2. Hyperlipidemia was observed in HFD-fed obese mice with elevated serum concentration of LDL, TG, free fatty acid, and TC. Free fatty acid, TC, TG, and LDL concentrations in serum were significantly reduced in FCME-treated groups (0.1–0.5%) to the levels of ND-fed mice. Accordingly, FCME treatment significantly lowered AI values in HFD-fed obese mice.

### 3.3. Effect of FCME on Endocrine Peptides and Liver Function Enzymes

**Figure 3:**
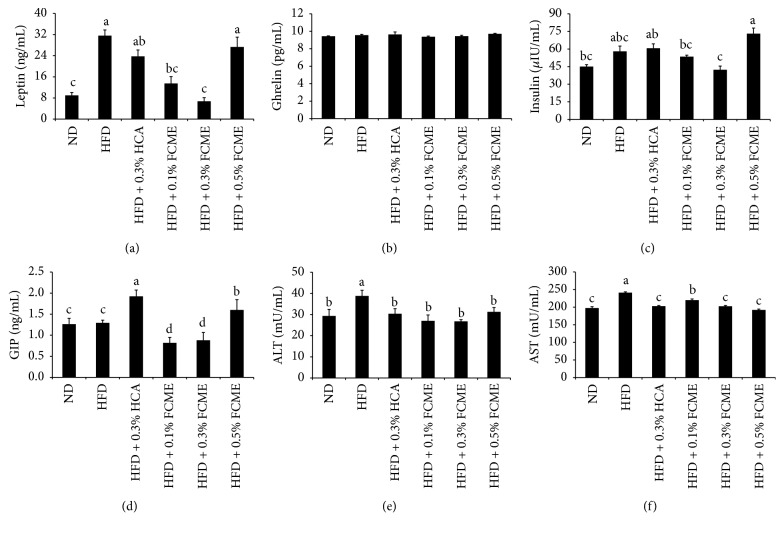
Effect of fermented* Cucurbita moschata* extract (FCME) on endocrine peptides and liver function enzymes of mice serum after 8-week feeding of ND, and 0.1%, 0.3%, and 0.5% of FCME-added high fat diet (HFD). (a) Leptin, (b) ghrelin, (c) insulin, (d) GIP, (e) ALT, and (f) AST. Different superscript letters are significantly different (*p* < 0.05) by ANOVA.

The impact of FCME on leptin, ghrelin, insulin, and GIP of mice serum are shown in Figure 3. Feeding of HFD increased the leptin level in mice which was markedly reduced with the supplementation of FCME (0.1–0.3%) in HFD. The levels of insulin and GIP in mice serum were also reduced significantly by feeding FCME-supplemented HFD. The serum concentrations of AST and ALT were pronouncedly high in HFD-fed obese mice (Figure 3). However, the concentrations of AST and ALT were reduced significantly in mice fed with FCME-supplemented HFD.

### 3.4. Effects of FCME on Hepatic Tissue and Adipocyte

**Figure 4:**
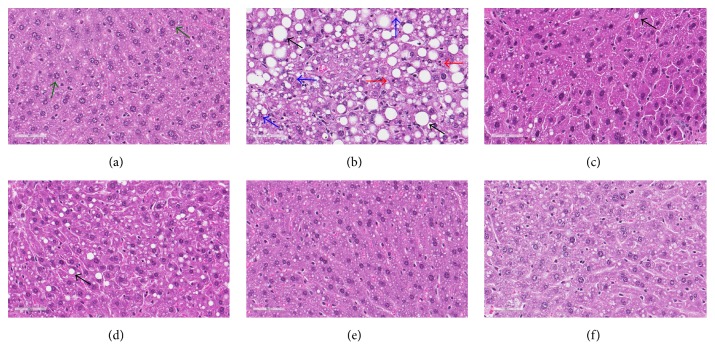
Haematoxylin and eosin staining of hepatic tissue (40x magnification). Mice fed normal diet (a); high fat diet (b); high fat diet with 0.3% HCA (c); high fat diet with 0.1% FCME (d); high fat diet with 0.3% FCME (e), and high fat diet with 0.5% FCME (f). Well-structured hepatocytes are indicated by green arrows in (a). Blue arrows in (b) indicate hepatocyte lipid inclusion (steatosis) and ballooning. Red arrows in (b) indicate lobular activity. Black arrows in (b), (c), and (d) indicate fat droplets.

The histopathological changes in liver tissues of mice associated with the feeding of HFD and FCME are summarized in Table 2 and Figure 4. The liver slices of ND-fed mice exhibited normal lobular architecture with well-structured hepatocytes. The feeding of HFD for 8 weeks ominously induced the steatosis, lobular activity, portal inflammation with fat droplets accumulation in hepatic tissues of mice. An increase in the accumulation of fat droplets was observed in the livers of the mice fed with HFD for 8 weeks compared to the ND-fed mice. The feeding of FCME-supplemented HFD to mice showed no or comparatively less accumulation of fat droplets in their hepatic tissues. Further, the addition of FCME in HFD significantly reduced steatosis, ballooning, hepatic inflammation, or lobular activity in mice (Table 2). The architecture and morphology of hepatocytes in FCME-treated mice were comparable to that observed in ND-fed mice.

**Figure 5:**
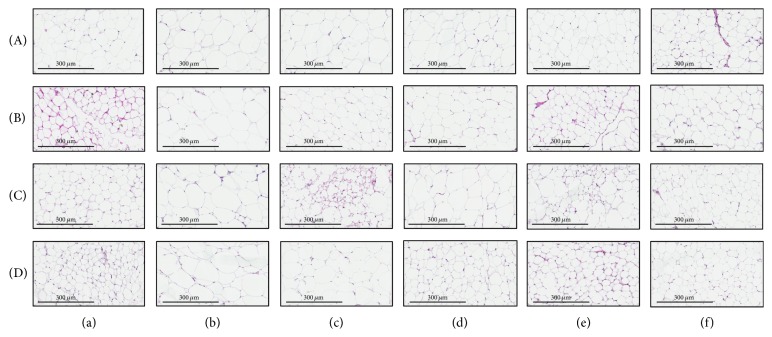
Haematoxylin and eosin staining of adipose tissues (10x magnification). In horizontal order, (A) epididymal, (B) mesenteric, (C) perirenal, and (D) subcutaneous adipose tissue. In vertical order, mice fed with (a) normal diet, (b) high fat diet, (c) high fat diet plus 0.3% HCA, (d) high fat diet plus 0.1% FCME, (e) high fat diet plus 0.3% FCME, and (f) high fat diet plus 0.5% FCME.

The effects of FCME on the size of adipocytes in adipose tissues collected from epididymal, mesenteric, perirenal, and subcutaneous regions are shown in Figure 5. The sizes of adipocyte in HFD-fed mice were markedly increased as compared to ND group. In contrast, feeding of FCME-supplemented HFD reduced the size of adipocytes in aforementioned adipose tissues of mice.

### 3.5. Effects of FCME on Gene Expression

The mRNA expressions of PPAR*γ*, C/EBP*α*, C/EBP_*β*_, C/EBP*γ*, and SREBP-1C were significantly upregulated in HFD-fed mice compared to ND-fed mice (Table 3). However, these genes were markedly downregulated in mice fed with FCME-supplemented HFD. The mRNA expressions of ACC1, FAS, LPL, and GLUT4 were downregulated in livers of HFD-fed mice as compared to ND-fed mice. There were no significant differences in gene expression pattern of above genes when mice fed HFD supplemented with FCME as compared to ND group.

### 3.6. Acute Oral Toxicity

The application of FCME (2000 mg/kg body weight) by oral route was safe. None of the animals showed behavioral manifestations of morbidity during the observation period. Furthermore, there were no remarkable differences in the body weight between control and treatment groups.

### 3.7. Compound Profile of FCME

The LC-MS and GC-MS analyses of FCME explored numerous types of compounds. The major identified compounds are listed in Table 4 with their reported biological activities. Mainly, surfactin derivatives are identified by LC-MS analysis, whereas pyrazine, tetramethyl, 2,3-butanediol, and some derivatives of 2,3-butanediol were detected and identified by GC-MS analysis.

## 4. Discussion

In this study, high fat diet-induced obesity model of mice was used which is considered as the most popular model among researchers because of its high similarity of mimicking the usual route of obesity episodes in human [17]. Moreover, feeding of HFD to animal can readily induce body weight gain and, hence, HFD-induced obesity model is considered as a reliable tool for studying obesity. A significant difference in the body weight gain was observed between HFD-fed mice and ND-fed mice, although the weekly food intake between these two groups of animals was not significantly different. This observation makes it clearly evident that the increased body weight gain in HFD-fed mice is not associated with the amount of food consumption. The feeding of FCME (0.1–0.5%) with HFD to animal reduced the body weight gain remarkably in comparison with the HFD only fed mice. This result suggests that the supplementation of 0.1% and 0.3% FCME can prevent the extra body weight gain, without inhibiting the general food consumption associated weight gain.

Obesity alters the endocrine and metabolic functions of adipose tissue and leads to increase in the release of hormones, fatty acids, and proinflammatory molecules that contribute to obesity associated complications [18]. Obesity can be described as a state of excessive growth of adipose tissue mass. Thus, the adipose tissue mass of animal was recorded for comparative study. A significant induction in the weight of subcutaneous, mesenteric, perirenal, and epididymal adipose tissues was observed in HFD-fed mice compared to ND-fed mice, which were remarkably suppressed in HFD+FCME-fed mice (0.1–0.3%) (Table 1). Subcutaneous and visceral abdominal adipose tissues are major types of adipose tissues. Visceral fat is composed of several adipose depots, including epididymal, mesenteric, and perirenal adipose tissues. Abdominal obesity is correlated with the increased risk of cardiovascular diseases and insulin resistance, whereas augmented subcutaneous fat is associated with a favorable plasma lipid profile [19]. Mesenteric adipose tissue is believed to play a central role in the development of obesity-related diseases [20]. Adiponectin and leptin are mainly available from subcutaneous adipocytes [19]. Certainly, the mean adipocyte mass of all types of adipose tissues was noticeably reduced in FCME-fed mice (Table 1). The major cellular component in adipose tissue is adipocyte; consequently, the mechanism that regulates the number and size of adipocyte has become a vital target in obesity research [21]. Excessive energy is stored as triglyceride in adipocytes and the expansion of adipocyte size (hypertrophy) means the elevation of intracellular lipid accumulation, which is dependent on the lipogenic activity of adipocyte [22]. The more influential aspect in the chronic situation of obesity is the increase in cell number (hyperplasia); however, the initial event of obesity development is hypertrophy [22].

Leptin and ghrelin are two hormones which are recognized to have major influence on energy balance. The adipocyte hormone that controls body weight by modulating food intake and energy expenditure is leptin [23]. The concentration of leptin is proportional to the percentage of body fat. Elevated levels of serum leptin were identified in obese people compared to nonobese people [24]. In the present study there was significant reduction in serum leptin in the HFD supplemented with 0.1% and 0.3% FCME as compared to HFD (Figure 3). This indicates that less body weight gain may be due to reduction of serum leptin [24]. Ghrelin controls the appetite and hunger by sending signal to brain that the subject is very hungry [25]. In this study, however; the levels of ghrelin were not significantly different among groups which indicate that the FCME does not influence the secretion and regulation of ghrelin.

In this study, apart from the reduction in body and adipose tissue weight, supplementation of FCME with HFD was observed to significantly attenuate the serum levels of total cholesterol (TC), free fatty acid (FFA), triglycerides (TG), atherogenic index (AI), and LDL. The basic mechanism behind the reduction in the level of TC, TG, FFA, LDL, and AI might be because of the breakdown of lipids by the components of the extract, thus modifying the altered lipid metabolism [26]. The reduction of TG, TC, LDL, and FFA in serum shows intensive clearance of circulating lipids. So reduction in lipoproteins and cholesterol levels further decreases complication and protects the cell from free radical induced damage [27]. Further, atherogenic index (AI) is considered as a marker for various cardiovascular disorders, and the AI value is directly proportional to the risk of developing cardiovascular disease and vice versa [28, 29]. Thus, reduced AI values observed in the FCME-treated groups (0.1–0.5%) are beneficial, because they possess lipid lowering properties with cardioprotective potential.

The ALT and AST have been the most commonly used parameters in laboratory testing for the evaluation of liver function, because these enzymes are cytoplasmic in location and are released into circulation after cellular damage [30]. The feeding of FCME- (0.1% and 0.3%) supplemented HFD significantly inhibited the diet-induced elevation of ALT and AST (Figure 3). In addition, histopathological investigation also showed the protective effect of FCME from inflammation and steatosis, which further consolidates the serum biochemical assay findings.

The feeding of HFD to mice significantly increased the mRNA expressions of PPAR_*γ*_, C/EBP_*α*_, C/EBP_*β*_, C/EBP_*γ*_, and SREBP-1c genes compared to ND-fed mice. There were no significant differences observed in mRNA expressions in mice fed HFD supplemented with FCME as compared to ND. In the past few decades, the role of PPAR_*γ*_ and SERBP-1c in the regulation of obesity and adipocyte differentiation has been highlighted and, hence, PPAR_*γ*_ agonists and antagonists of synthetic or herbal origin have gained wide commercial popularity as therapeutic agents [31]. Moreover, PPAR_*γ*_ regulates the expression of adipocyte genes such as adipocyte-fatty acid binding protein (A-FABP) [32]. SREBP-1c controls the expression of lipogenic genes such as ACC and FAS [33]. SREBP-1c also activates the C/EBP_*β*_ promoter and insulin-mediated transcriptional factors [24]. Significant upregulation of PPAR_*γ*_, C/EBP_*α*_, and SERBP-1c mRNA expressions in the present study is in accordance with the previous reports [34, 35]. We observed tendencies for reduced SREBP-1c in the FCME-fed groups compared to the HFD group; however, we did not find any significant reduction in mRNA expression of FAS, the rate-limiting enzyme of fatty acid synthesis in the liver (Table 3). Moreover, LPL, which is related to fat intake, did not differ among the HFD only group and HFD-plus-FCME-fed groups. Therefore, it shows that the antiobesity effect of FCME is responsible for the decreased expression of fatty acid metabolism-related genes rather than reduced fatty acid synthesis and fat intake in the liver.

The expression of another PPAR_*γ*_-mediated transcriptional factor, ACC1, was not changed. The expression and function of insulin-responsive GLUT4 are regulated by the PPAR_*γ*_ and free fatty acids [36]. The one of the main glucose transporters is GLUT4; it was reported that the changes in the expression of this gene were more related to type 2 diabetes and insulin resistance rather than obesity [37]. In this study, the GLUT4 mRNA expression in the liver tissue is increased significantly by FCME. Moreover, the level of insulin elevated in HFD group was significantly reduced by FCME supplementation (Figure 3) except for 0.5% FCME. Hyperinsulinemia is considered as a biomarker of insulin resistance, and it is frequently accompanied by obesity [3]. It is expected that the antidiabetic activity of FCME can be attributed to the regulation of GLUT4 and insulin.

Probiotic* Bacillus subtilis* and lactic acid bacteria are reported in many recent studies for their preventive effect on obesity and metabolic syndrome [11, 13]. These probiotic bacteria produce 2,3-butanediol [38]. In this study, 2,3-butanediol is identified and quantified as the major compound (about 73%) of fermented FCME by GC-MS analysis. Our result is supported by the identification of lactate, acetate, and 2,3-butanediol in the growth medium as the major anaerobic fermentation products by using high-performance liquid chromatography [39]. Butanediol induces a reduction of cerebral glycolytic rate and affects the cerebral energy metabolism [40]. The cerebral energy metabolism has a very close relationship with systemic energy metabolism [41]. Surfactins, which are identified from the FCME by LC-MS/MS analysis, have been reported earlier for antidiabetic, antilipidemic, and antihypercholesterolemic properties [7, 13]. Thus, the antiobesity effect of FCME might be because of the presence of butanediol and surfactins in the extract.

In conclusion, the coadministration of FCME (0.1–0.3%) with HFD restored the altered lipid profile and hepatic injury in the studied diet-induced obese mice model. The control of this extract on the regulation of these genes led to decreased lipid level, body weight, fat mass, adipocyte size, and hepatic abnormalities. This study is the first scientific report that provides convincing evidence for the relevance of fermented* C. moschata* as a food with antiobesity properties. These results suggest that the fermented* C. moschata* extract can be a promising candidate for the prevention of obesity.

## Figures and Tables

**Table 1:** Effect of fermented *Cucurbita moschata* extract on organs weight.

Name of organ	ND	HFD	HFD + 0.3% HCA	HFD + 0.1% FCME	HFD + 0.3% FCME	HFD + 0.5% FCME
Liver (g)	0.95 ± 0.05^b^	1.16 ± 0.05^a^	1.13 ± 0.05^a^	0.91 ± 0.04^b^	0.94 ± 0.05^b^	1.04 ± 0.04^ab^
Kidney (Right) (g)	0.18 ± 0.01	0.18 ± 0.01	0.18 ± 0.01	0.17 ± 0.01	0.17 ± 0.01	0.17 ± 0.01
Kidney (Left) (g)	0.17 ± 0.01^ab^	0.18 ± 0.01^a^	0.17 ± 0.01^ab^	0.16 ± 0.01^ab^	0.15 ± 0.01^b^	0.16 ± 0.01^ab^
Spleen (g)	0.08 ± 0.00^ab^	0.09 ± 0.00^a^	0.07 ± 0.01^ab^	0.07 ± 0.00^b^	0.07 ± 0.00^ab^	0.07 ± 0.01^b^
Mesenteric adipose tissue (g)	0.40 ± 0.02^b^	1.01 ± 0.11^a^	0.80 ± 0.05^a^	0.57 ± 0.05^b^	0.49 ± 0.08^b^	0.81 ± 0.10^a^
Epididymal adipose tissue (g)	0.69 ± 0.06^c^	1.86 ± 0.17^a^	1.77 ± 0.07^a^	1.41 ± 0.11^b^	1.01 ± 0.15^c^	1.84 ± 0.10^a^
Subcutaneous adipose tissue (g)	0.36 ± 0.04^c^	1.08 ± 0.12^a^	0.95 ± 0.06^a^	0.65 ± 0.06^b^	0.46 ± 0.10^bc^	0.91 ± 0.11^a^
Perirenal adipose tissue (g)	0.29 ± 0.03^c^	1.03 ± 0.11^a^	0.94 ± 0.05^a^	0.57 ± 0.05^b^	0.46 ± 0.10^bc^	0.89 ± 0.07^a^
Total adipose tissue (g)	1.74 ± 0.12^c^	4.98 ± 0.48^a^	4.46 ± 0.20^a^	3.20 ± 0.23^b^	2.42 ± 0.41^bc^	4.45 ± 0.34^a^

ND: normal diet; HFD: high fat diet; HCA: hydroxycitric acid; FCME: fermented *Cucurbita moschata* extract. Values are presented as mean ± SE (*n* = 10); values not having the same superscript letter are significantly different (*p* < 0.05) by ANOVA.

**Table 2:** Histological evaluation of liver of mice treated with fermented *Cucurbita moschata* extract.

Groups	Steatosis	Ballooning	Portal inflammation	Lobular activity	Fatty changes (lipidosis)
ND	0.67 ± 0.58^d^	0.00 ± 0.00^d^	0.00 ± 0.00^b^	0.00 ± 0.00^c^	0.33 ± 0.58^b^
HFD	4.67 ± 0.58^a^	4.00 ± 1.00^a^	3.33 ± 1.15^a^	2.67 ± 1.53^a^	5.00 ± 0.00^a^
HFD + 0.3% HCA	2.00 ± 0.00^bc^	2.00 ± 1.00^b^	1.00 ± 1.00^b^	1.33 ± 0.58^abc^	0.67 ± 0.58^b^
HFD + 0.1% FCME	2.67 ± 0.58^b^	1.67 ± 0.58^bc^	1.00 ± 1.00^b^	1.67 ± 0.58^ab^	1.00 ± 1.00^b^
HFD + 0.3% FCME	1.00 ± 1.00^cd^	0.67 ± 0.58^cd^	0.33 ± 0.58^b^	0.67 ± 0.58^bc^	0.33 ± 0.58^b^
HFD + 0.5% FCME	0.33 ± 0.58^d^	0.00 ± 0.00^d^	0.67 ± 0.58^b^	0.00 ± 0.00^bc^	0.67 ± 0.58^b^

Duncan's multiple range test. 0: absent; 1: few; 2: mild; 3: moderate; 4: severe; and 5: extremely severe. ND: normal diet; HFD: high fat diet; HCA: hydroxycitric acid; FCME: fermented *Cucurbita moschata *extract; values not having the same superscript letter are significantly different (*p* < 0.05) by ANOVA.

**Table 3:** Relative expressions of adipogenic and lipogenic genes of mice liver tissue after 8-week treatment with fermented *Cucurbita moschata* extract.

Gene	ND	HFD	HFD + 0.3% HCA	HFD + 0.1% FCME	HFD + 0.3% FCME	HFD + 0.5% FCME
PPAR-*γ*	1.00 ± 0.00^bc^	1.62 ± 0.14^a^	1.21 ± 0.09^abc^	0.83 ± 0.07^c^	0.76 ± 0.05^c^	1.38 ± 0.09^ab^
SREBP-1C	1.00 ± 0.00^b^	1.77 ± 0.02^a^	1.18 ± 0.03^b^	1.08 ± 0.10^b^	1.07 ± 0.09^b^	1.04 ± 0.13^b^
CEBP-*α*	1.00 ± 0.00^b^	1.58 ± 0.04^a^	1.03 ± 0.11^b^	1.40 ± 0.14^ab^	1.32 ± 0.08^ab^	1.37 ± 0.15^ab^
CEBP-*β*	1.00 ± 0.00^c^	1.76 ± 0.06^a^	1.39 ± 0.05^b^	1.03 ± 0.13^c^	1.29 ± 0.06^bc^	1.30 ± 0.03^bc^
CEBP-*γ*	1.00 ± 0.00^a^	1.27 ± 0.04^a^	1.22 ± 0.03^a^	0.61 ± 0.05^b^	1.00 ± 0.10^a^	0.99 ± 0.05^a^
ACC1	1.00 ± 0.00	1.06 ± 0.06	0.88 ± 0.02	0.93 ± 0.01	1.09 ± 0.03	1.01 ± 0.04
FAS	1.00 ± 0.00	1.18 ± 0.06	1.22 ± 0.08	1.34 ± 0.12	1.47 ± 0.09	1.32 ± 0.19
GLUT4	1.00 ± 0.00^c^	0.39 ± 0.01^d^	1.07 ± 0.03^bc^	1.46 ± 0.08^ab^	0.94 ± 0.04^c^	1.53 ± 0.09^a^
LPL	1.00 ± 0.00	1.10 ± 0.08	0.94 ± 0.09	0.92 ± 0.02	0.84 ± 0.06	0.87 ± 0.08

ND: normal diet; HFD: high fat diet; HCA: hydroxycitric acid; FCME: fermented *Cucurbita moschata* extract. PPAR-*γ*: peroxisome proliferator-activated receptor-*γ*; C/EBP-*α*: CCAAT/enhancer binding protein-*α*; C/EBP-*β*: CCAAT/enhancer binding protein-*β*; C/EBP-*γ*: CCAAT/enhancer binding protein-*γ*; FAS: fatty acid synthase; LPL: lipoprotein lipase; GLUT4: glucose transporter 4; SREBP-1C: sterol regulatory element-binding protein-1C; ACC1: acetyl-CoA carboxylase 1; values not having the same superscript letter are significantly different (*p* < 0.05) by ANOVA.

**Table 4:** List of compounds identified from fermented *Cucurbita moschata* extract by LC-MS and GC-MS analysis.

LC-MS					
RT (min)	Name of compound	*m*/*z* ([M + H]^+^)	△ppm	Pharmacological properties	References

24.74	Surfactin C13	1022.6766	1.142	Antiviral, antimycoplasma, antibacterial, antihypercholesterolemia, inflammation suppressor	[7]
25.17	Surfactin C14	1036.36925	1.271	Antiviral, antimycoplasma, antibacterial, antihypercholesterolemia, inflammation suppressor	[7]

GC-MS					
RT (min)	Name of compound		Area (%)	Pharmacological properties	References

4.26	Cinnamyl cinnamate		6.53	-	-
7.07	Ammonia		5.66	-	-
23.64	Acetic Acid		3.68	-	-
24.01	Pyrazine, tetramethyl		1.13	Cardioprotective, antihypertensive, vasodilator, anti-inflammatory, anticoagulant, free radical-scavenging	[8]
25.97	2,3-Butanediol		45.86	Psychoactive	[8]
39.66	2,3-Butanediol, formamide		26.90	Effective against asthma	[9]
40.40	Methyl propyl ether		1.58	-	-

## Supplementary Materials

Supplementary Materials Supplementary Table 1: compositions of experimental diet; the source of the feeds is Dyets Inc. (Bethlehem, PA, USA). Supplementary Table 2: base sequences of adipogenic and lipogenic primers.
