# Supplementary material for: Enhancement of Lipid Metabolism and Hepatic Stability in Fat-Induced Obese Mice by Fermented Cucurbita moschata Extract
Source: Evid Based Complement Alternat Med. 2018 Mar 13;2018:3908453. doi: 10.1155/2018/3908453 (PMC5872621; doi:10.1155/2018/3908453)
Supplement: Supplementary Materials — Supplementary Table 1: compositions of experimental diet; the source of the feeds is Dyets Inc. (Bethlehem, PA, USA). Supplementary Table 2: base sequences of adipogenic and lipogenic primers. [file 3908453.f1.pdf]

Supplementary Results:

Supplementary Table 1: Compositions of experimental diet. The source of the feeds is Dyets Inc., (Bethlehem, PA, USA).

Ingredients	Normal diet (ND) AIN-76A diet # 100000		High-fat diet (HFD) AIN-76A diet # 101556	
	g	kcal	g	kcal
Beef tallow	0	0	400	3600
Casein	200	720	200	720
Cellulose	50	0	50	0
Choline bitartrate	2	0	2	0
Corn oil	50	450	0	0
Methionine	3	12	3	12
Salt mixture	35	30.8	35	30.8
Starch	150	540	150	540
Sucrose	500	2000	150	600
Vitamin mixture	10	39	10	39
Total	1000	3791.8	1000	5541.8

Supplementary Table 2: Base sequences of adipogenic and lipogenic primers.

**Gene Targets**	**Forward Primer (5' – 3')**	**Reverse Primer (5' – 3')**	**Product Size (bp) / Accession No.**	**Reference**
PPAR-γ_1	CGGTTTCAGAAGTGCCTTGC	CCGCCAACAGCTTCTCCTT	90	[1]
C/EBP-α	GAGCCGAGATAAAGCCAAACA	CGGTCATTGTCACTGGTCAACT	74	[2]
C/EBP-β	AAGCTGAGCGACGAGTACAAGA	GTCAGCTCCAGCACCTTGTG	NM_009883	[3]
C/EBP-γ	TCCACGACTCCTGCCATGTA	GCGGCCATGGAGTCAATG	70	[4]
FAS	GGCTCAGCATGGTCGCTT	CTCCCGCCAGCTGTCATT	232	[5]
LPL	GATTTCTCTGTACGGCACAGTGG	TTTGTGGAAACCTCGGGC	68	[6]
GLUT4	TTGGTACCTACGCTTTGCAGC	CGGTTAGAGCGCATCAGTCTC	68	[6]
SREBP-1C	GTAGCGTCTGCACGCCCTA	CTTGGTTGTTGATGAGCTGGAG	89	[5]
ACC1	GGATGGTTTGGCCTTTCACA	TTTTCTTTCTGTCTCGACCTTGTTT	76	[6]
β-actin	TGACAGGATGCAGAAGGAGAT	CTCCTGCTTGCTGATCCACAT	-	-

PPAR-γ, peroxisome proliferator-activated receptor-γ; C/EBP-α, CCAAT/enhancer binding protein-α; C/EBP-β, CCAAT/enhancer binding protein-β; C/EBP-γ, CCAAT/enhancer binding protein-γ; FAS, fatty acid synthase; LPL, lipoprotein lipase; GLUT4, glucose transporter 4; SREBP-1C, sterol regulatory element-binding protein-1C; ACC1, acetyl-CoA carboxylase

Supplementary Reference:

[1] C. Schachtrup, T. E. Scholzen, V. Grau, et al., "L-FABP is exclusively expressed in alveolar macrophages within the myeloid lineage: Evidence for a PPARα-independent expression" *The International Journal of Biochemistry & Cell Biology*, vol. 36, no. 10, pp. 2042–2053, 2004.

[2] N. Sue, B. H. A. Jack, S. A. Eaton, et al., "Targeted Disruption of the Basic Krüppel-Like Factor Gene (Klf3) Reveals a Role in Adipogenesis" *Molecular and Cellular Biology*, vol. 28, no. 12, pp. 3967–3978, 2008.

[3] K. Yu, D. Mo, M. Wu, et al., "Activating transcription factor 4 regulates adipocyte differentiation via altering the coordinate expression of CCATT/enhancer binding protein β and peroxisome proliferator-activated receptor γ" *The FEBS Journal*, vol. 281, no. 10, pp. 2399–2409, 2014.

[4] M. Y. Wang, P. Grayburn, S. Chen, M. Ravazzola, L. Orci, R. H. Unge, "Adipogenic capacity and the susceptibility to type 2 diabetes and metabolic syndrome" *Proceedings of the National Academy of Sciences*, vol. 105, no. 16, pp. 6139–6144, 2008.

[5] A. Gomori, A. Ishihara, M. Ito, et al., "Blockade of MCH1 receptor signalling ameliorates obesity and related hepatic steatosis in ovariectomized mice" *British Journal of Pharmacology*, vol. 151, no. 6, pp. 900–908, 2007.

[6] M. A. Reza, M. A. Hossain, D. Damte, W. S. Jo, W. H. Hsu, S. C. Park, "Hypolipidemic and Hepatic Steatosis Preventing Activities of the Wood Ear Medicinal Mushroom *Auricularia auricula-judae* (Higher Basidiomycetes) Ethanol Extract In Vivo and In Vitro" *International Journal of Medicinal Mushrooms*, vol. 17, no. 8, pp. 723–734, 2015.
